# *Rhodiola rosea* suppresses thymus T-lymphocyte apoptosis by downregulating tumor necrosis factor-α-induced protein 8-like-2 in septic rats

**DOI:** 10.3892/ijmm.2015.2241

**Published:** 2015-06-10

**Authors:** MING-WEI LIU, MEI-XIAN SU, WEI ZHANG, LIN-MING ZHANG, YUN-HUI WANG, CHUAN-YUN QIAN

**Affiliations:** 1 Department of Emergency, The First Affiliated Hospital of Kunming Medical University, Kunming, Yunnan 650032, P.R. China; 2 Department of Emergency, The Second Affiliated Hospital of Kunming Medical University, Kunming, Yunnan 650101, P.R. China; 3 Department of Neurology, The First Affiliated Hospital of Kunming Medical University, Kunming, Yunnan 650032, P.R. China

**Keywords:** *Rhodiola rosea*, tumor necrosis factor-α-induced protein 8-like-2, T lymphocyte, apoptosis, caecal ligation and puncture, mice

## Abstract

In recent years, several studies have shown that *Rhodiola rosea* can enhance cellular immunity and humoral immune function in mice, and thus, it has become a research hotspot. However, its underlying mechanism of action has remained elusive. The present study investigated whether *Rhodiola rosea* was able to downregulate the expression of tumor necrosis factor-α-inducible protein 8-like 2 (TIPE2), thereby inhibiting the expression of apoptotic genes, attenuating T-lymphocyte apoptosis and improving immunity in septic mice. A mouse model of caecal ligation and puncture (CLP)-induced sepsis was established, and animals in the treatment group were pre-treated with an intraperitoneal injection of *Rhodiola rosea* extract, while animals in the control group and sham-operated group were injected with an equivalent amount of normal saline. TIPE2, B-cell lymphoma 2 (Bcl-2), Fas and Fas ligand (FasL) mRNA and protein levels in thymic T cells were determined using reverse transcription quantitative polymerase chain reaction and western blot analysis, respectively. Furthermore, the thymus T-lymphocyte apoptosis rate, thymus T-lymphocyte count and thymus T-lymphocyte sub-sets were assessed using flow cytometry. Levels of T-helper cell type 1 (Th1) cytokines [Interleukin (IL)-2, IL-12 and interferon (IFN)-γ] and Th2 cytokines (IL-4 and IL-10) were determined using ELISA. The results showed that, compared to that in the CLP group, the expression of TIPE2, Fas and FasL in the treatment group was significantly decreased, while the expression of Bcl-2 was increased (P<0.05). The thymus lymphocyte count in the CLP group was significantly higher compared with that in the treatment group (P<0.05). Furthermore, the apoptotic rate of thymus T-lymphocytes in the treatment group was significantly lower than that in the CLP group (P<0.05). In addition, treatment with *Rhodiola rosea* rescued decreased in the counts of the CD3^+^ T and CD4^+^ T sub-sets of thymus T lymphocytes in the CLP group (P<0.05), while not affecting the increased levels of Th2 cytokines (IL-4 and IL-10) in the CLP group compared with those in the control groups. In addition, the Th1 cytokines (IL-12, IL-2 and IFN-γ) were significantly increased (P<0.05) in the CLP group, and treatment with *Rhodiola rosea* led to further increases. The thymus index of septic mice treated with *Rhodiola rosea* as well as their survival rate were improved as compared with those in the CLP group. These findings suggested that *Rhodiola rosea* has protective effects against sepsis by decreasing apoptosis, increasing Th1 cytokines and enhancing the host’s immunity via the regulation of TIPE2 expression.

## Introduction

Sepsis or systemic inflammatory response syndrome (SIRS) is caused by infection (1) and is a type of complication that is commonly observed in trauma, major surgery, burns and other clinical diseases (2), which can lead to septic shock and multiple organ dysfunction syndrome (MODS), one of the leading causes of mortality in patients (3). Several studies have shown that immunosuppression is implicated in sepsis (4), which is mainly attributed to the accelerating apoptosis of lymphocytes (mainly including B lymphocytes and T-helper cells) and dendritic cells (5). Hotchkiss *et al* (4) found that lymphocyte apoptosis is associated with the severity of sepsis. Apoptosis of great a number of lymphocytes in sepsis renders the body in an immunosuppressive state, which cannot effectively regulate the specific immune response against a pathogenic infection, resulting in multiple organ failure and mortality (4).

Tumor necrosis factor (TNF)-α-inducible protein 8-like 2 (TIPE2), a newly identified protein, is essential for maintaining immune homeostasis (6). TIPE2 shares considerable sequence homology with members of the TNF-1-inducible protein 8 (TNFAIP8) family, which is thought to regulate cellular and immune homeostasis. TNFAIP8, the first identified member of this family, is able to enhance cell survival and inhibit apoptosis (7,8). TIPE2 is a recently identified negative regulator of innate and adaptive immunity and is preferentially expressed in lymphoid tissues (9).

*Rhodiola rosea*, a rhodiola plant of the *Crassulaceae* family, is a perennial herb or shrub plant that is widely distributed in China. *Rhodiola rosea*, a common Tibetan medicine, is mainly used in the treatment of conditions including hot phlegm cough, haemoptysis, injuries, burns, leucorrhoea and diabetes (10). Modern pharmacological studies have shown that *Rhodiola rosea* has the following effects: Anti-ageing, anti-fatigue, anti-oxidant, anti-tumor and anti-viral effects, as well as enhancement of learning memory, resistance to microwave radiation, enhancement of immunity, protection of the viscera, enhancement of the physique, improvement of haematopoietic function, resistance to fatigue, lowering of blood sugar and prevention of altitude sickness (11,12). *Rhodiola rosea* is highly effective in the treatment of diabetes (12), ischaemic heart disease (13) and free radical injury during cerebral ischaemia-reperfusion (14,15). Salidroside is one of the major active components of *Rhodiola rosea* (16). In recent years, *in vitro* experiments as well as animal experiments have indicated that *Rhodiola rosea* can enhance cellular immunity and humoral immune function (17).

The present study aimed to determine whether *Rhodiola rosea* extract is able to enhance the expression of TIPE2, reduce thymus T-lymphocyte apoptosis and improve immunity. By observing changes in thymus T lymphocytes, TIPE2 and immune cells in mice with sepsis which were pre-treated with *Rhodiola rosea* extract, the present study aimed to elucidate the immune regulatory mechanisms of *Rhodiola rosea*.

## Materials and methods

### Preparation of plant extract

An ethanolic extract of the *Rhodiola rosea* root was used in the present study. The *Rhodiola rosea* root used for the extraction was obtained from Baoxing Biotechnologies Co., Ltd. (Guangzhou, China) and was authenticated by a plant taxonomist. The plant root (50 g) was first dried and ground to a coarse plant powder, which was then extracted by boiling with 500 ml of 70% ethanol twice for 2 h each. The extract was concentrated under reduced pressure, precipitated with ethanol and finally spray dried using a WD800ASL microwave extraction apparatus (Galanz Co., Shunde, China) to yield a reddish-brown powder. The yield of the *Rhodiola rosea* extract was ~3–5% (w/w).

### Mice

Thirty-two nine-week-old male BALB/c mice weighing 22–30 g were purchased from the Experimental Animal Center of Kunming Medical University (Kunming, China) and were acclimated to laboratory conditions for one week prior to the experiment. All experiments were approved and conducted in accordance with the guidelines of the Animal Care Committee of Kunming Medical University (Kunming, China). The experimental procedures were approved by the Ethics Committee of the Institute of Yunan University of Traditional Chinese Medicine (TCM; Kunming, China). The initial body weight of the mice showed no significant difference among the four groups in the present study. They were maintained in a well-ventilated, controlled room at 20°C with a 12-h light/dark cycle with access to water and food *ad libitum*.

### Generation of TIPE2-deficient mice

Next, 2.2-kb and 5.0-kb TIPE2 genomic fragments were amplified using polymerase chain reaction (PCR) and cloned into the *Xho*I/*Nhe*I and *Not*I/*Sal*I sites of the pOSDUPDE vector (a gift from Dr X.P. Zhong, Department of Pediatrics, Duke University, Durham, NC, USA), respectively. As has been previously described (18), PCR was conducted with Lightcycler (Roche Diagnostics, Mannheim, Germany). All reactions were performed using a SYBR Green PCR mix (Takara Biotechnology, Otsu, Japan), according to the following thermal profile: denaturation at 95°C for 30 sec, followed by 40 cycles of 95°C for 5 sec, 60°C for 30 sec and 72°C for 30 sec. The primer sequences were obtained from Sangon Biological Engineering Co., Ltd. (Shanghai, China) and were as follows: TIPE2 forward, 5′-GGAACATCCAAGGCAAGACTG-3′ and reverse, 5′-AGCACCTCACTGCTTGTCTCATC-3′. Gene-specific amplifications were demonstrated with melting curve and gel-migration analyses. Gels were stained using SYBR Gold Nucleic Acid Gel Stain (Invitrogen Life Technologies, Carlsbad, CA, USA) in 1X TBE (dilution, 1:16,000) buffer for 40 min and bands were visualized by UV transillumination on a Bio-Rad Gel Doc 2000 (Bio-Rad, Ivry-sur-Seine, France). As has been previously described (19), wild-type (WT) C57BL/6 mice, which carry a TIPE2 gene-null mutation, were generated by backcrossing TIPE2^−/−^ mice to WT C57BL/6 mice for 12 generations. TL1 embryonic stem (ES) cells from 129S6/SvEvTac mice (Shanghai Laboratory Animal Center of the Chinese Academy of Science, Shanghai, China) were transfected with the targeting vector and subjected to positive and negative selection using G418 (Guangzhou Huawei Chemical Co., Ltd, Guangzhou, China) and ganciclovir (Jena Biosciences, Jena, Germany), respectively (20). Two ES cell clones, in which the *TIPE2* gene (including exons 1 and 2) was replaced by the neomycin resistance gene cassette, were identified using Southern blot analyses. For the southern blot analysis, 7–10 *µ*g of isolated DNA were digested with *Eco*RI and *Nru*I and separated on a 0.8% agarose/Tris acetate EDTA (TAE) gel. Following DNA transfer, the membranes were hybridized using the TIPE2-specific genomic probe StB12.3 (21). The mutant ES cells were injected into four-day-old C57BL/6J mouse blastocysts (Shanghai Laboratory Animal Center of the Chinese Academy of Science). The resulting chimeric male offspring were crossed with 129S6/SvEvTac females for germ-line transmission. Unless otherwise indicated, all mice used in the present study were of the 129S6/SvEvTac background. Age- and gender-matched littermates were used as controls. The mice were housed in the Experimental Animal Center of Kunming Medical University under pathogen-free conditions. All procedures used were pre-approved by the Institutional Animal Care and Use Committee.

### Reagents

Flow cytometry was performed by using a FACSCalibur flow cytometer from Becton-Dickinson (Franklin Lakes, NJ, USA). The SHIMADZU analytical balance (type, AUW120D) was obtained from Shanghai Ruifang Instrument Co., Ltd. (Shanghai, China). The ALPU cryogenic refrigerator (type, DW-60L398) was obtained from Bio-Rad Laboratories Inc. (Hercules, CA, USA). The TRIzol kit and fetal calf serum (FCS) were purchased from Invitrogen Life Technologies. The reverse transcription (RT) reaction kit was obtained from Takara Biotechnology. TRIzol and the electrophoresis reagents were purchased from ProMag Co., Ltd. (Ningbo, China). The PCR amplification reagent kit and DNA ladder marker were obtained from Sangon Biological Engineering Co., Ltd.. β-actin was obtained from Santa Cruz Biotechnology Inc. (Dallas, TX, USA); TNF-α, interleukin (IL)-6, IL-10, interferon (IFN)γ and IL-12 ELIS) kits were obtained from the Science and Technology Development Center of the People’s Liberation Army General Hospital (Beijing, China). Rabbit anti-mouse Fas was from Cell Signaling Technology (Beverly, MA, USA), and fas ligand (FasL, 1:200) and B-cell lymphoma 2 (Bcl-2, 1:200) polyclonal antibodies were obtained from Wuhan Boster Biological Technology, Ltd. (Wuhan, China). Fluorescently labeled antibodies, including a CD3-peridinin chlorophyll, CD4-fluorescein isothiocyanate (FITC) and CD8-allophycocyanin cocktail (all 1:200) were purchased from BD Biosciences. RPMI-1640 medium and EDTA disodium salt were obtained from Sigma-Aldrich (St Louis, MO, USA).

### Animal model of sepsis

Sepsis was induced by caecal ligation and puncture (CLP), as previously described (22). Briefly, the mice were anaesthetised by isoflurane inhalation, and a 2-cm ventral midline abdominal incision was performed. The cecum was then exposed, ligated immediately distal to the ileocecal valve to avoid intestinal obstruction, punctured twice with an 18-guage needle, and returned to the abdominal cavity. The incision was then closed in layers. Sham-operated animals underwent the same procedure, with the exception that the cecum was neither ligated nor punctured. The animals were resuscitated with 3 ml/100 g body weight normal saline, which was administered subcutaneously immediately after surgery.

### Groupings and treatments in vivo

According to a random number table, 80 mice were randomly divided into four groups: Normal control group, TIPE2-deficient group, sham-operated group (sham group), sepsis model group (model group) and *Rhodiola rosea* extract treatment group (treatment group), with 20 mice per group. The model group and treatment group were induced by CLP. Animals in the treatment group were administered intraperitoneal injections of *Rhodiola rosea* extract (at 50 mg/kg body weight) 8 h prior to surgery, while the normal control, sham and control groups were given the same volume of normal saline.

### Sample preparation

After the animals in each group were anaesthetised with ether 24 h following CLP, the right internal carotid artery was isolated. Blood was extracted (5 ml) and the blood was centrifuged (10,000 × g for 5 min) to collect the supernatant. The blood was dispensed into two sterile tubes, sealed with sealing glue and placed into the freezer at −20°C for examination. The thymus was compressed in Teflon tissue homogenizers (BILON-08; Shanghai Bilon Instrument Manufacturing Co. Ltd., Shanghai,China), and the resulting single-cell suspensions were pelleted at 300 xg, subjected to hypotonic shock for red cell removal, washed in RPMI-1640 containing 10% fetal bovine serum (FBS) and quantified. Macrophages were removed from the cell suspensions by plastic adherence (23) using a Kenker 24 cell adherent culture plate (Longchuan Jiaxing Bio Technology Co., Ltd., Jiaxing, China) in pre-warmed RPMI-1640 containing 5% FCS at 37°C for 1 h in a CO_2_ incubator. The T lymphocytes were purified on nylon wool columns [Nylon Fiber Column T (L-Type); Wako Pure Chemical Industries, Ltd., Wako, Japan] according to the method of Julius *et al* (24). The thymus tissues were removed for histopathological and immunofluorescence analyses.

### RT-quantitative (q)PCR

T cells were homogenized in TRIzol™ reagent using a Mixer 301 (Invitrogen Life Technologies). Total RNA was extracted according to the manufacturer’s instructions. Total RNA (1 *µ*g) was incubated with 200 units Moloney Murine Leukemia Virus reverse transcriptase in buffer containing 50 mmol/l Tris HCl (pH 8.3) (Shanghai Jiang Lai Biological Technology Co., Ltd., Shanghai, China), 75 mmol/l KCl (Shanghai Jiang Lai Biological Technology Co., Ltd.), 3 mmol/l MgCl_2_ (Sangon Biological Engineering Co., Ltd.), 20 units RNase inhibitor (Sangon Biological Engineering Co., Ltd.), 1 *µ*mol/l poly(dT) oligomer (Becton Dickinson) and 0.5 mmol/l each dNTP (Becton Dickinson) in a final volume of 20 *µ*l. The reaction mixture was incubated at 42°C for 1 h and then at 94°C for 5 min to inactivate the enzyme. A total of 80 *µ*l diethyl pyrocarbonate treated water was added to the reaction mixture prior to storage at 70°C. Real-time PCR was performed as previously described (12). Briefly, RNA samples were treated with DNase and subjected to quantitative PCR, which was performed with the ABI Prism 7000 sequence detection system (Applied Biosystems, Fisher Termo Scientific, Waltham, MA, USA) using SYBR-Green I dye (Gibco-BRL, Invitrogen Life Technologies), and the threshold cycle numbers were obtained using ABI Prism 7000 SDS software version 1.0 (Applied Biosystems). Conditions for amplification were 1 cycle at 94°C for 5 min followed by 40 cycles of 94°C for 30 sec, 58°C for 30 sec and 72°C for 45 sec. The primer sequences (Sangon Biological Engineering Co., Ltd.) used in the present study are shown in Table I. β-actin was used as an endogenous control. The DNA products of the RT-PCR reactions were separated by 4% SDS-PAGE in the same buffer. The polyacrylamide gels were dried and scanned using the ImageQuant densitometer (LI-COR Biosciences, Lincoln, NE, USA) and the comparative Ct (2^−ΔΔCt^) method for relative expression was performed (25). The PCR products were subjected to melting curve analysis, and the standard curve was used to confirm the correct amplification.

### Western blot analysis

T lymphocytes were washed twice with ice-cold phosphate-buffered saline (PBS) and lysed in lysis buffer [25 mM Tris-HCl pH 7.5 (Shanghai Jiang Lai Biological Technology Co., Ltd.), 150 mM NaCl (Shanghai Jiang Lai Biological Technology Co., Ltd.), 1% Nonidet P-40 (Sangon Biological Engineering Co., Ltd.), 5 mM sodium pyrophosphate (Xinxiang Huaxing Chemical Co., Ltd., Xinxiang, China), 1 mM sodium orthovanadate (Selleck Chemicals, Houston, TX, USA), 10 mM sodium fluoride (Shanghai Kunxin Chemical Technology Co., Ltd., Zibo, China), 10% glycerol (Solarbio Science & Technology, Beijing, China), 1 mM phenylmethylsulfonyl fluoride (Chemical Reagent Co., Ltd., Shanghai, China), 25 *µ*g/ml leupeptin (Shanghai Hufeng Chemical Co., Ltd., Shanghai, China), 25 *µ*g/ml aprotinin (Shanghai Hufeng Chemical Co., Ltd.) and 2 *µ*g/ml pepstatin (Shanghai Hufeng Chemical Co., Ltd.)] and incubated for 30 min on ice. The proteins were separated using 4–12% gradient SDS-PAGE and transferred onto polyvinylidene difluoride membranes. The primary antibodies used included rabbit anti-TIPE2 monoclonal antibody (1:200), rabbit anti-Fas monoclonal antibody (1:200), rabbit anti-FasL monoclonal antibody (1:200), mouse anti-Bcl-2 monoclonal antibody (1:200) and were purchased from Wuhan Boster Biological Technology, Ltd. The secondary antibodies used were horseradish peroxidase (HRP)-linked goat anti-rabbit immunoglobulin G (IgG) (1:4,000 dilution; Amersham Pharmacia Biotech, Piscataway, NJ, USA) and sheep anti-mouse IgG-HRP (1:8,000 dilution; Amersham Pharmacia Biotech). The blots were visualized by enhanced chemiluminescence (ECL) using a Pierce-ECL western blotting substrate (Pierce Biotechnology Inc., Rockford, IL, USA) and a Johnson enhanced chemiluminescence immunoassay analyzer (Shanghai Qian Jin Industrial Co., Ltd., Shanghai, China). Immunoreactive proteins were visualized using horseradish peroxidase-conjugated secondary antibodies and chemiluminescence (Millipore, Billerica, MA, USA). The relative expression intensity of the proteins was determined as the target band grey value normalized to the β-actin band grey value. The experiment was repeated three times.

### Flow cytometric detection of the apoptotic rate of thymic T cells

Thymus T lymphocytes were fixed with 70% ethanol and treated with RNase (Heino Chemical Co., Ltd., Zhuhai, China). Next, the nuclei were stained with propidium iodide (PI; Abcam, Cambridge, MA, USA) and FITC-Annexin V (BD Pharmingen, San Diego, CA, USA). The DNA content was measured using a FACSCalibur flow cytometer and CellQuest software (Becton Dickinson). Ten thousand cells were quantified in all of the assays. Apoptotic cells were quantified as the percentage of cells stained with Annexin V.

### Flow cytometric T-lymphocyte quantification and terminal deoxynucleotidyl transferase dUTP nick end labeling (TUNEL)

For flow cytometric analysis, the thymus T lymphocytes were fixed in 70% ethanol overnight at 4°C. Thymus T lymphocytes were washed in PBS with 0.1% bovine serum albumin. The cells were incubated with 1 U ml of RNase A (DNase free; BD Pharmingen) and 10 *µ*g ml-1 of PI overnight at room temperature in the dark. The cells were analyzed using a FACSCalibur flow cytometer, and CellQuest software (Becton Dickinson) was used to determine the relative DNA content based on the presence of red fluorescence. TUNEL analysis was performed to determine the apoptotic rate in the thymus according to the manufacturer’s instructions (Roche Applied Science, Basel, Switzerland). Six micrographs were randomly selected and the numbers of healthy or apoptotic thymocytes were counted. The percentage of apoptotic cells was defined as the percentage of the percentage of TUNEL-positive cells.

### Thymus index assay

Mice were sacrificed by cervical dislocation and the thymus was removed. The surface of the thymus was dried of blood using a filter paper and weighed using a one hundred thousandth electronic balance. The thymus index was calculated as follows: Thymus index = thymus weight/body weight ×100%.

### Determination of thymus T-lymphocyte sub-sets (CD3^+^, CD4^+^ and CD8^+^)

Thymus T lymphocyte suspensions in cold PEB buffer [PBS supplemented with 2 mM EDTA and 0.5% BSA (JRH Biosciences, Lenexa, KS, USA)] were incubated with supermagnetic microbeads (Microbead™ M530; Dongguan Sanhe Chemical Co., Ltd., Dongguan, China) conjugated to anti-mouse CD3, anti-mouse CD4 or anti-mouse CD8 monoclonal antibodies at 4°C for 15 min. The cells were washed twice and loaded onto magnetic separation columns [Nylon Fiber Column T (L-Type); Dako Cytomation, Carpinteria, CA, USA). The columns were washed three times with cold PEB buffer, and the CD3^+^, CD4^+^ or CD8^+^ T cells were then eluted. After purification, the cells were consistently >95% viable, as assessed using trypan blue exclusion (Takara Biotechnology). Fluorescence-assisted cell sorting analysis was performed using a Becton Dickinson LSR analyse (Becton Dickinson) and anti-mouse FITC-CD3 (BD Biosciences), anti-mouse FITC-CD4 (Sigma-Aldrich) and anti-mouse FITC-CD8 (BD Biosciences).

### Th1 and Th2 cytokine assays

Th1 cytokines (IL-2, IL-12 and IFNγ) and Th2 cytokine (IL-4 and IL-10) were determined using commercially available ELISA kits (Pierce Biotechnology Inc.), according to the manufacturer’s instructions.

### Histopathological examination

Thymus tissue was fixed in 10% formalin for 24 h followed by dehydration. The thymus was embedded in paraffin wax, sectioned into 5-*µ*m slices and stained with Mayer’s hematoxylin and eosin (Merck Millipore, Darmstadt, Germany). Micrographs of the lung sections were captured with a CX21 light microscope (Olympus, Tokyo, Japan).

### Survival curves

Another 45 mice were divided into the sham operation group, CLP group and CLP plus *Rhodiola rosea* extract group (n=15 per group) to determine the survival rate. The treatments were identical to those of the previous experiments (3). Observation was commenced from the start of *Rhodiola rosea* extract treatment, while the endpoint was set at 120 h after *Rhodiola rosea* extract treatment.

### Statistical analysis

Values are expressed as the mean ± standard error of the mean. For comparisons among multiple groups, one-way or two-way analysis of variance followed by Bonferroni’s post-hoc test was used to determine significant differences. Differences between two groups were tested using Student’s t-test. P<0.05 was considered to indicate a statistically significant difference between values. All statistical analyses were carried out using SPSS version 13.0 for Windows (SPSS Inc., Chicago, IL, USA).

## Results

### Downregulation of TIPE2 decreases Fas and FasL protein expression and apoptosis, while increasing Bcl-2 in thymus T-lymphocytes of septic mice

**Figure 1:**
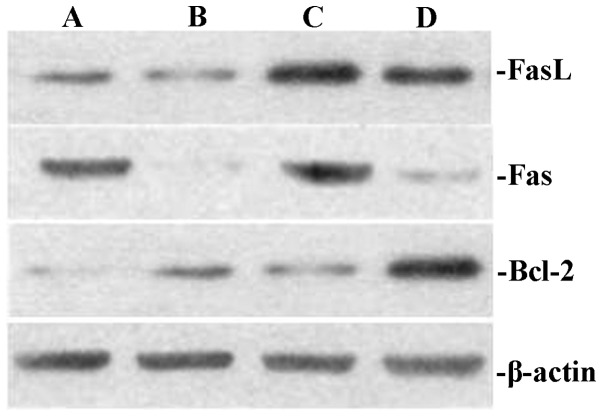
Effect of TIPE2 on the expression of Bcl-2, Fas and FasL in thymus T cells from septic mice. Groups of mice were induced by caecal ligation and puncture for 24 h. Representative western blot showing the levels of Bcl-2, Fas and FasL protein expression in thymus T cells in septic mice. Control group (non-septic group): (A) TIPE2-deficient mice; (B) wild-type mice; (C) TIPE2-deficient mice; (D) wild-type mice. TIPE2, tumor necrosis factor-α-inducible protein 8-like 2; Bcl-2, B-cell lymphoma 2; FasL, Fas ligand.

**Figure 2:**
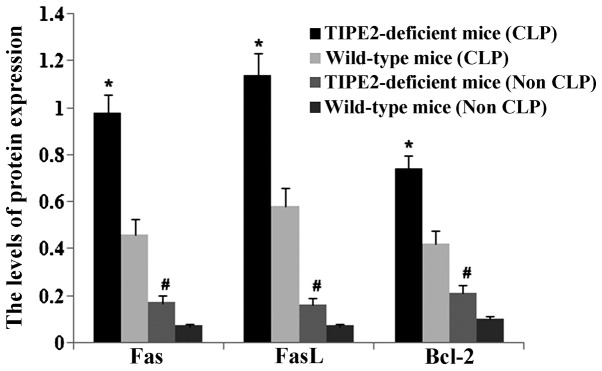
TIPE2 increases Bcl-2 protein expression and blocks Fas and FasL protein expression in thymus T cells from septic mice. Groups of mice were induced by caecal ligation and puncture for 24 h. Results were obtained by densitometric analysis of western blots of Bcl-2, Fas and FasL protein. Values are expressed as the mean ± standard deviation of three independent experiments. Non-septic group: ^*^P<0.05 vs. wild-type mice; septic group: ^#^P<0.05 vs. wild-type mice. TIPE2, tumor necrosis factor-α-inducible protein 8-like 2; Bcl-2, B-cell lymphoma 2; FasL, Fas ligand.

**Figure 3:**
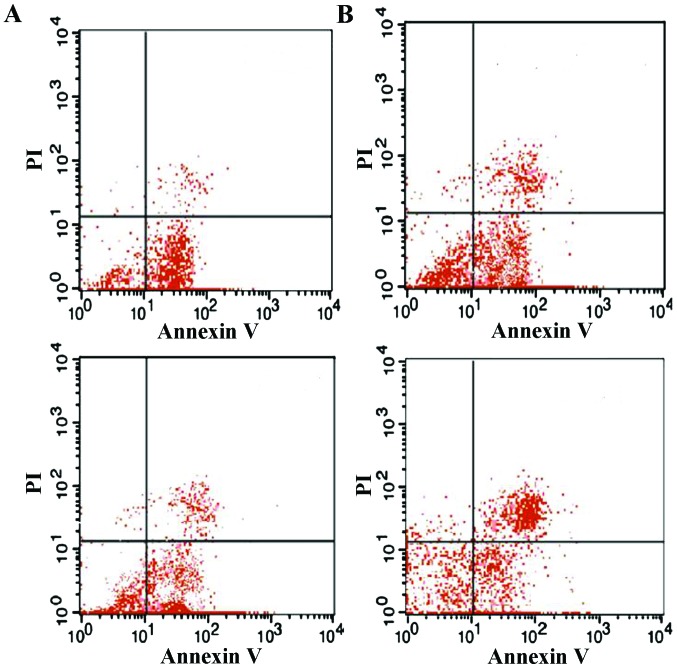
Effect of TIPE2 on the thymus T-lymphocyte apoptosis rate in septic mice. Groups of mice were challenged by caecal ligation and puncture. Following 24 h, the thymus T-lymphocyte apoptosis rate was assessed using flow cytometry. Representative dot plots show T-lymphocyte apoptosis in thymus T cells from septic mice. (A) Non-septic group: top panel, wild-type mice; bottom panel, TIPE2-deficient mice. (B) Septic group: top panel, wild-type mice; bottom panel, TIPE2-deficient mice. TIPE2, tumor necrosis factor-α-inducible protein 8-like 2; PI, propidium iodide.

**Figure 4:**
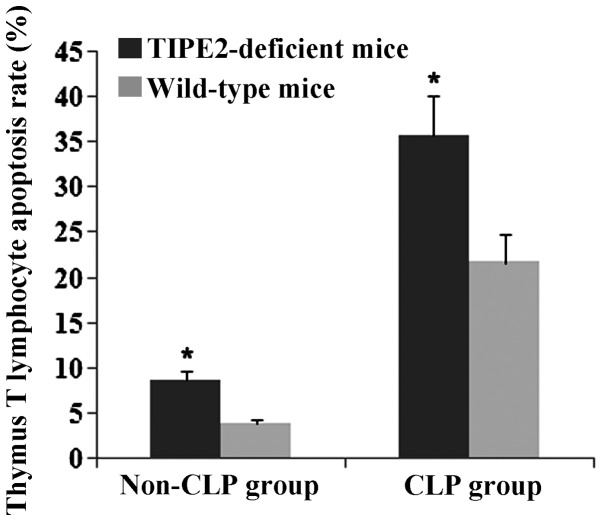
TIPE2 increases the thymus T-lymphocyte apoptosis rate in septic mice. Groups of mice were challenged by caecal ligation and puncture. 24 h later, the thymus T-lymphocyte apoptosis rate was measured using flow cytometry followed by quantitative evaluation. Values are expressed as the mean ± standard error of the mean of three experiments. ^*^P<0.05 vs. wild-type mice. TIPE2, tumor necrosis factor-α-inducible protein 8-like 2.

The present study investigated the effects of TIPE2 on Bcl-2, Fas and FasL expression in thymic T cells in septic mice. After 12 h, the CLP model was established in TIPE2-deficient mice and WT mice. Bcl-2, FaS and FasL protein expression in thymic T cells were determined using western blot analysis. The thymus T-lymphocyte apoptosis rate was assessed using flow cytometric analysis. As shown in Figs. 1–4, Fas and FasL protein expression and T-lymphocyte apoptosis were markedly increased in TIPE2-deficient mice compared with those in WT mice (P<0.05). In addition, Bcl-2 protein expression was significantly decreased in TIPE2-deficient mice compared with those in WT mice (P<0.05).

### Rhodiola rosea extract attenuates CLP-induced increases in TIPE2, FaS and FasL expression as well as decreases in Bcl-2 expression

**Figure 5:**
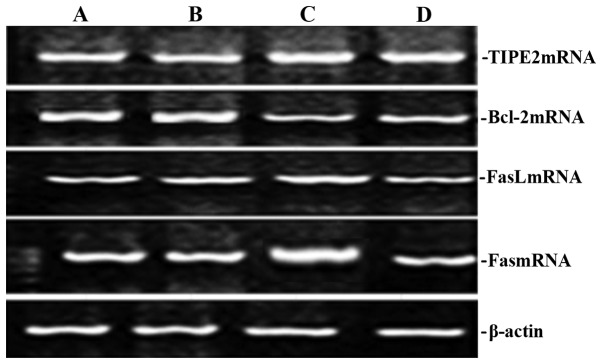
Effect of *Rhodiola rosea* extract on the expression of TIPE2, Bcl-2, Fas and FasL mRNA in the thymus of septic mice. Groups of mice were induced by caecal ligation and puncture for 24 h. Representative gels of reverse transcription polymerase chain reaction products showing the levels of TIPE2, Bcl-2, Fas and FasL mRNA expression in rats. (A) Normal control group; (B) sham operation group; (C) control group; (D) treatment group. TIPE2, tumor necrosis factor-α-inducible protein 8-like 2; Bcl-2, B-cell lymphoma 2; FasL, Fas ligand.

**Figure 6:**
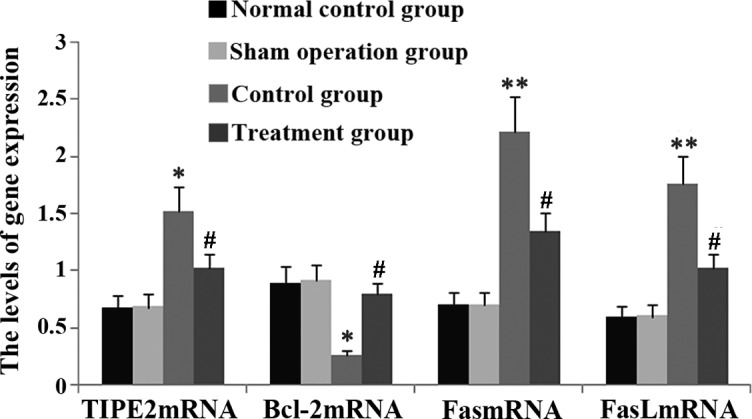
*Rhodiola rosea* extract inhibits the expression of TIPE2, Fas and FasL mRNA in the thymus of septic mice. Groups of mice were treated with *Rhodiola rosea* extract and 8 h later challenged by caecal ligation and puncture for 24 h. Expression of TIPE2, Bcl-2, Fas and FasL mRNA was determined using reverse transcription polymerase chain reaction and quantified by densitometric analysis. Values are expressed as the mean ± standard error of the mean. ^*^P<0.05, ^**^P<0.01 vs. sham operation group and normal control group; ^#^P<0.05 vs. control group. TIPE2, tumor necrosis factor-α-inducible protein 8-like 2; Bcl-2, B-cell lymphoma 2; FasL, Fas ligand.

**Figure 7:**
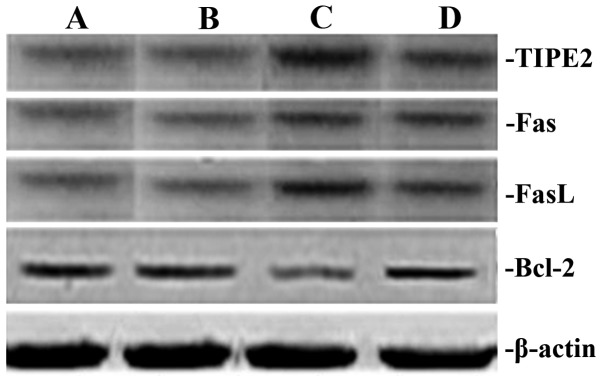
Effect of *Rhodiola rosea* extract on the expression of TIPE2, Bcl-2, Fas and FasL in thymus T cells from septic mice. Groups of mice were treated with *Rhodiola rosea* extract and 8 h later challenged by caecal ligation and puncture for 24 h. Representative western blots show the levels of TIPE2, Bcl-2, Fas and FasL expression. (A) Normal control group; (B) sham-operated group; (C) control group; (D) treatment group. TIPE2, tumor necrosis factor-α-inducible protein 8-like 2; Bcl-2, B-cell lymphoma 2; FasL, Fas ligand.

**Figure 8:**
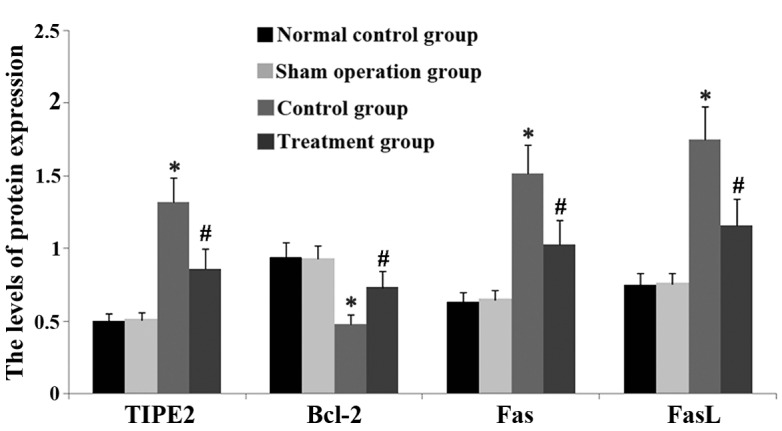
*Rhodiola rosea* extract suppresses the expression of TIPE2, Bcl-2, Fas and FasL in thymus T cells from septic mice. Groups of mice were treated with *Rhodiola rosea* extract and 8 h later challenged by caecal ligation and puncture for 24 h. The expression of TIPE2, Bcl-2, Fas and FasL protein was assessed using western blot analysis and quantified by densitometric analysis. Values are expressed as the mean ± standard deviation of three experiments. ^*^P<0.05, P<0.01 vs. sham-operated group and normal control group; ^#^P<0.05 vs. control group. TIPE2, tumor necrosis factor-α-inducible protein 8-like 2; Bcl-2, B-cell lymphoma 2; FasL, Fas ligand.

To assess the potential effect of *Rhodiola rosea* on the expression of TIPE2, Bcl-2, FaS and FasL in CLP-induced mice, their protein and mRNA levels were determined using RT-qPCR and western blot analyses, respectively, in thymic T cells of mice at 24 h after CLP challenge, which was performed 8 h after pre-treatment with *Rhodiola rosea* extract. As shown in Figs. 5–8, the expression of TIPE2, FaS and FasL was markedly enhanced, while the expression of Bcl-2 was significantly decreased at 24 h after CLP challenge as compared with those in the control groups (all P<0.05). Of note, pre-treatment with *Rhodiola rosea* extract decreased the expression of TIPE2, FaS and FasL, and increased the expression of Bcl-2 as compared with that in the CLP group (P<0.05), therefore attenuating the CLP-induced changes.

### Rhodiola rosea extract reduces the apoptotic rate of thymus T lymphocytes in septic mice

**Figure 9:**
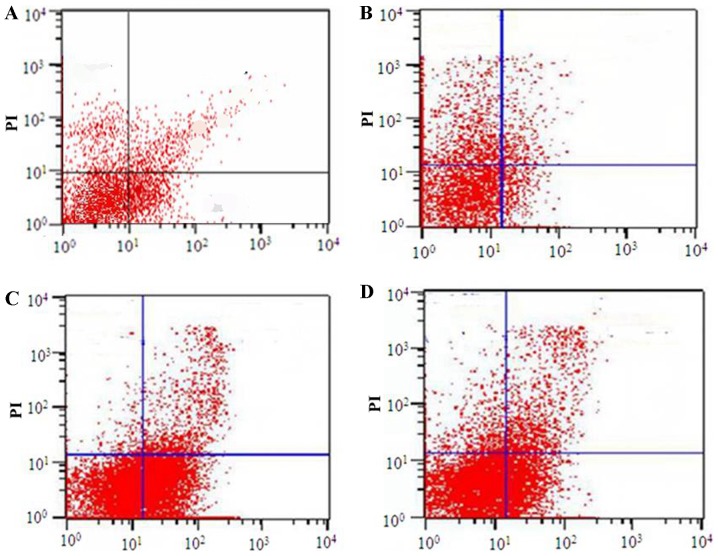
Effect of *Rhodiola rosea* extract on the thymus T-lymphocyte apoptosis rate in septic mice. Groups of mice were treated with *Rhodiola rosea* extract and 8 h later challenged by caecal ligation and puncture for 24 h. The thymus T-lymphocyte apoptosis rate was measured using flow cytometry. Representative dot plots showing thymus T-lymphocyte apoptosis in septic mice. (A) Normal control group; (B) sham-operated group; (C) control group; (D) treatment group. PI, propidium iodide.

**Figure 10:**
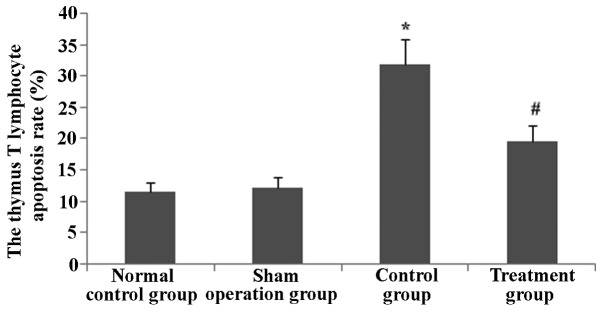
*Rhodiola rosea* extract decreases the thymus T-lymphocyte apoptosis rate in septic mice. Groups of mice were treated with *Rhodiola rosea* extract and 8 h later challenged by caecal ligation and puncture for 24 h. The apoptotic rate was determined by statistical evaluation of the flow cytometric results. Values are expressed as the mean ± standard error of the mean. ^*^P<0.01 vs. sham operation group; ^#^P<0.05 vs. control group.

**Figure 11:**
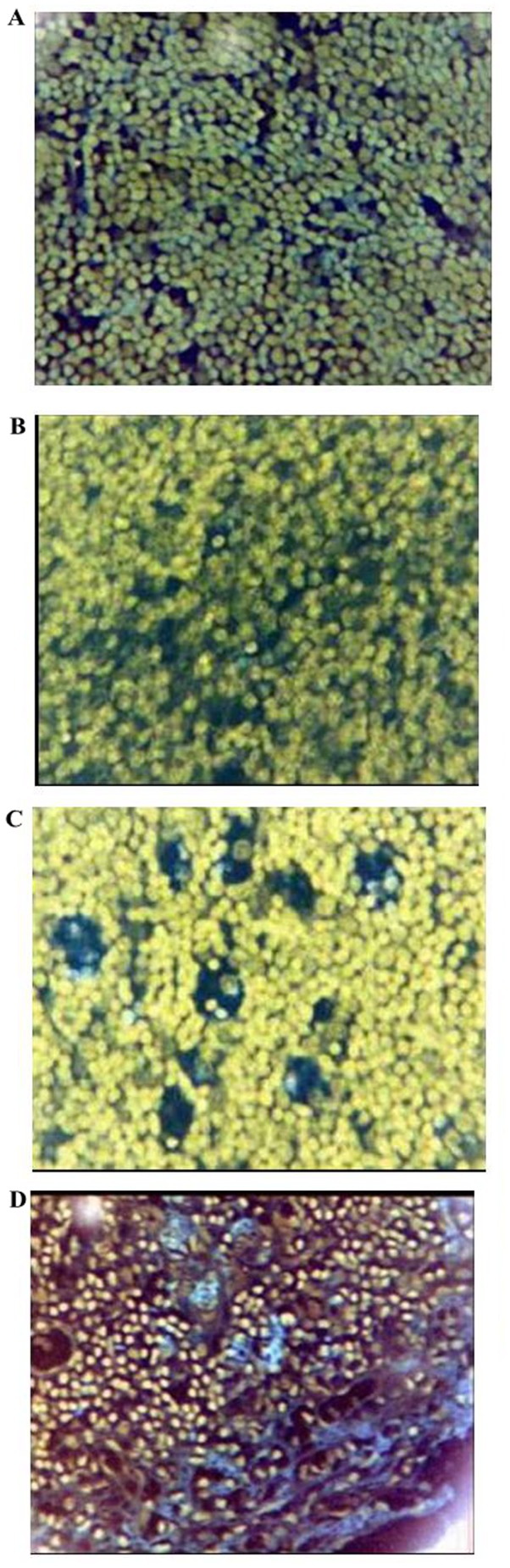
*Rhodiola rosea* extract inhibits thymus T-lymphocyte apop-tosis in mice. Groups of mice were treated with *Rhodiola rosea* extract and 8 h later challenged by caecal ligation and puncture for 24 h, followed by TUNEL analysis. Representative TUNEL images indicating thymus T-lymphocyte apoptosis in mice at 24 h after administration of *Rhodiola rosea* extract. (A) Normal control group; (B) sham-operated group; (C) control group; (D) treatment group. TUNEL, terminal deoxynucleotidyl transferase dUTP nick end labeling; magnification, x200.

**Figure 12:**
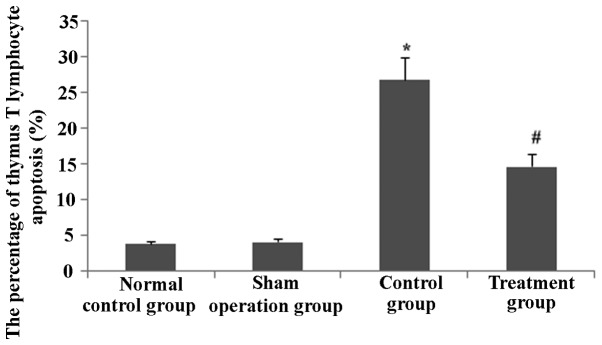
*Rhodiola rosea* extract inhibits thymus T-lymphocyte apoptosis in mice according to TUNEL analysis. The percentage of TUNEL-stained thymus T-lymphocytes of mice 24 h following caecal ligation and puncture performed 8 h following administration of *Rhodiola rosea* extract was determined by statistical evaluation of the histochemical images. Values are expressed as the mean ± standard deviation of three experiments. ^*^P<0.05 compared with untreated model group; ^#^P<0.05 vs. control group. TUNEL, terminal deoxynucleotidyl transferase dUTP nick end labeling.

To determine the effect of *Rhodiola rosea* extract on thymus T-lymphocyte apoptosis in septic mice, flow cytometry and TUNEL assays were performed. After the groups of mice were induced with CLP, the apoptotic rate of thymus lymphocytes was markedly enhanced. Of note, in the group pre-treated with *Rhodiola rosea* extract, the apoptotic rate of thymus lymphocytes was markedly decreased as compared with that in the CLP group (all P<0.05). Thus, *Rhodiola rosea* extract markedly decreased the apoptotic rate of thymus lymphocytes (Figs. 9–12).

### Rhodiola rosea extract attenuates decreases in the thymus T-lymphocyte count in septic mice

**Figure 13:**
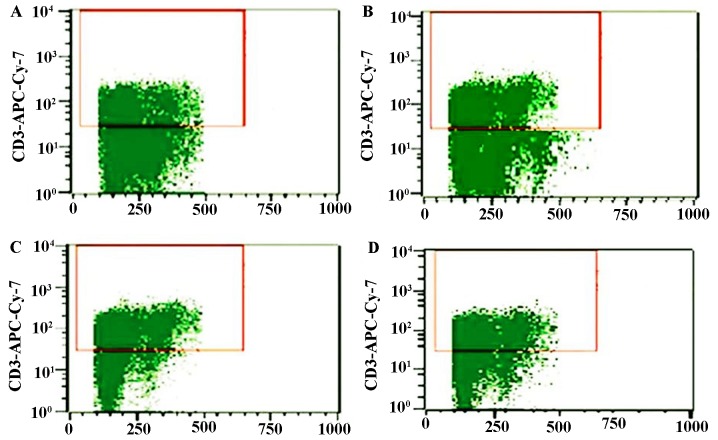
Effect of *Rhodiola rosea* extract on the thymus T-lymphocyte count in septic mice. Groups of mice were treated with *Rhodiola rosea* extract and 8 h later challenged by caecal ligation and puncture for 24 h. The thymus T-lymphocyte count was measured using flow cytometry. Representative flow cytometry dot plots showing the count of thymus T lymphocytes in mice. (A) Normal control group; (B) sham-operated group; (C) control group; (D) treatment group. The populations in the red windows are the count of thymus T lymphocytes.

**Figure 14:**
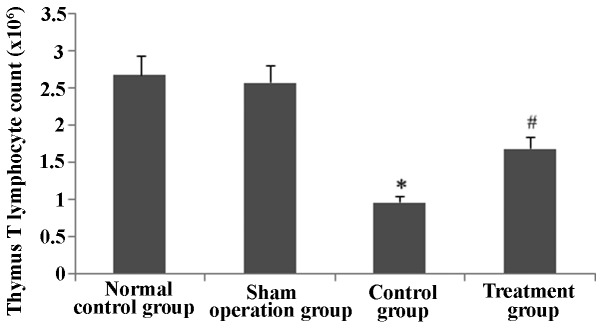
*Rhodiola rosea* extract increases the thymus T-lymphocyte count in septic mice. Groups of mice were pre-treated with *Rhodiola rosea* extract and challenged by caecal ligation and puncture for 24 h. Thymus T-lymphocytes were quantified using flow cytometry. Values are expressed as the mean ± standard error of the mean. ^*^P<0.05 vs. sham-operated group; ^#^P<0.05 vs. control group.

As sepsis stimulated thymus T lymphocyte apoptosis, the thymus T-lymphocyte count was determined. As shown in Figs. 13 and 14, the thymus T-lymphocyte counts were markedly decreased in CLP-induced mice at 24 h (P<0.05); however, in the group pre-treated with *Rhodiola rosea* extract, the thymus T lymphocyte count was significantly increased (P<0.05).

### Rhodiola rosea extract rescues the decreases in CD3^+^, CD4^+^, CD8^+^ and CD4^+^/CD8^+^ T-lymphocyte sub-sets in septic mice

**Figure 15:**
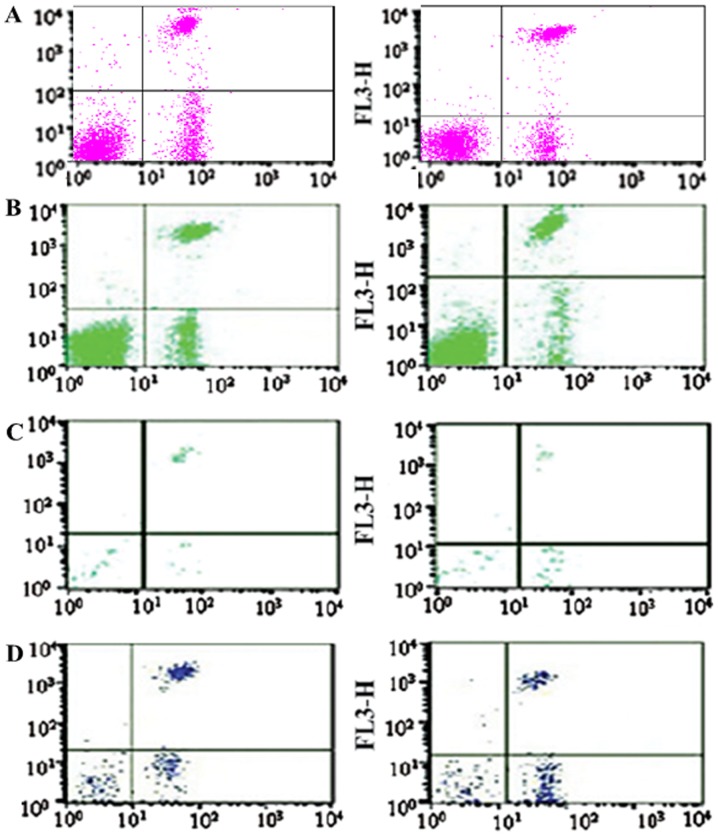
Effect of *Rhodiola rosea* extract on thymus T-lymphocyte sub-sets in septic mice. Groups of mice were treated with *Rhodiola rosea* extract and 8 h later challenged by caecal ligation and puncture for 24 h. Thymus T-lymphocyte sub-sets were determined using flow cytometry. Representative flow cytometry dot plots showing the percentage of thymus T-lymphocyte subsets. (A) Normal control group; (B) sham-operated group; (C) control group; (D) treatment group.

**Figure 16:**
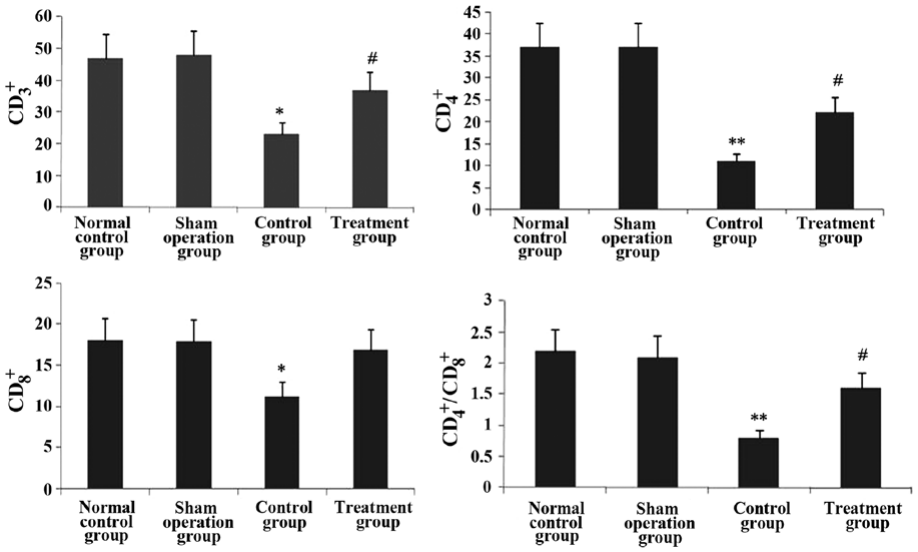
Effect of *Rhodiola rosea* extract on thymus T-lymphocyte sub-sets in septic mice. Groups of mice were treated with *Rhodiola rosea* extract and 8 h later challenged by caecal ligation and puncture for 24 h. Thymus T-lymphocyte sub-sets were measured using flow cytometry. Statistical quantification of CD3^+^, CD4^+^, CD8^+^ and CD4^+^/CD8^+^ in rats. Values are expressed as the mean ± standard error of the mean. ^*^P<0.05, ^**^P<0.01 vs. sham operation group and normal control group; ^#^P<0.05, P<0.01 vs. control group.

The effect of *Rhodiola rosea* extract on thymus T-lymphocyte sub-sets in septic mice was evaluated. As shown in Figs. 15 and 16, the CD3^+^, CD4^+^, CD8^+^ and CD4^+^/CD8^+^ T-lymphocyte sub-sets were markedly decreased in septic mice (all P<0.05). However, pre-treatment with *Rhodiola rosea* extract significantly increased the CD3^+^, CD4^+^, CD8^+^ and CD4^+^/CD8^+^ T-lymphocyte sub-sets (all P<0.05).

### Rhodiola rosea extract enhances increases of Th1 cytokines in the plasma of septic mice, while not affecting increases in Th2 cytokines

**Figure 17:**
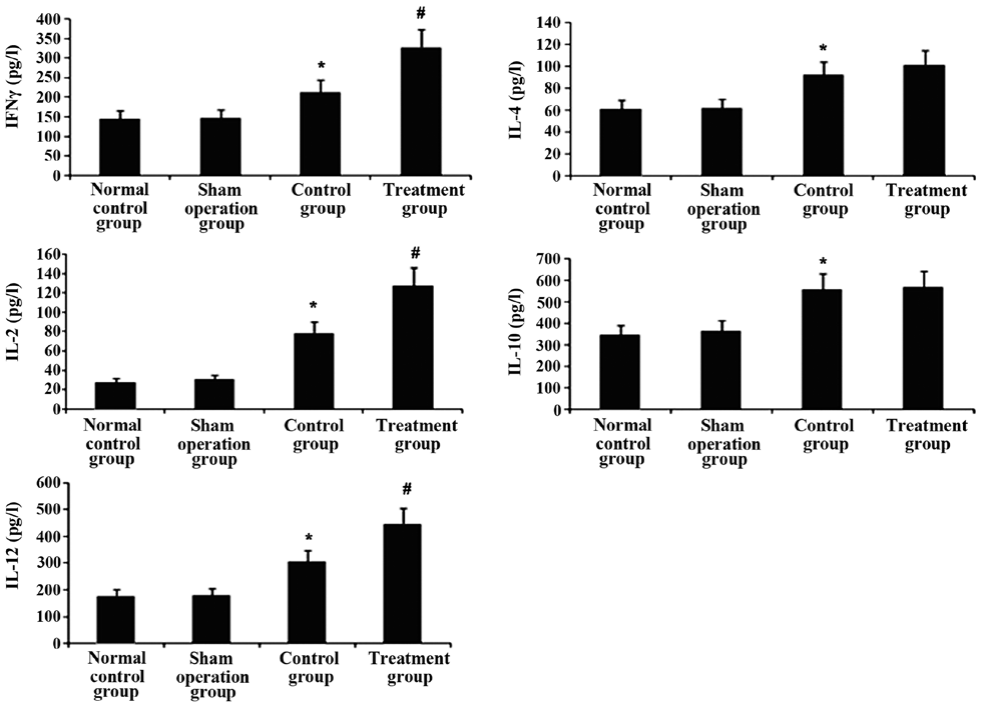
Effect of *Rhodiola rosea* extract on Th1/Th2 cytokines in the plasma of septic mice. Groups of mice were treated with *Rhodiola rosea* extract and 8 h later challenged by caecal ligation and puncture for 24 h. Levels of Th1 cytokines (IFNγ, IL-2 and IL-12) and Th2 cytokines (IL-4 and IL-10) in the plasma were measured using ELISA, and the ratio of IFNγ/IL-4 was determined. Values are expressed as the mean ± standard error of the mean. ^*^P<0.05, ^**^P<0.01 vs. sham operation group and normal control group. ^#^P<0.05, P<0.01 vs. control group. IL, interleukin; Th, T-helper cell; IFN, interferon.

To study the effects of *Rhodiola rosea* extract on Th1/Th2 cytokines, levels of Th1 cytokines (IFNγ, IL-2 and IL-12) and Th2 cytokines (IL-4 and IL-10) in plasma were measured using ELISA. As shown in Fig. 17, Th1 and Th2 cytokines were markedly enhanced at 24 h after CLP challenge. Of note, pre-treatment with *Rhodiola rosea* extract led to further increases in Th1 cytokines (IFNγ, IL-2 and IL-12; all P<0.05), while Th2 cytokine levels were not significantly altered compared with those in the model (sepsis) group. These results indicated that treatment with *Rhodiola rosea* extract attenuated the host’s immunity.

### Histopathological changes of the thymus

**Figure 18:**
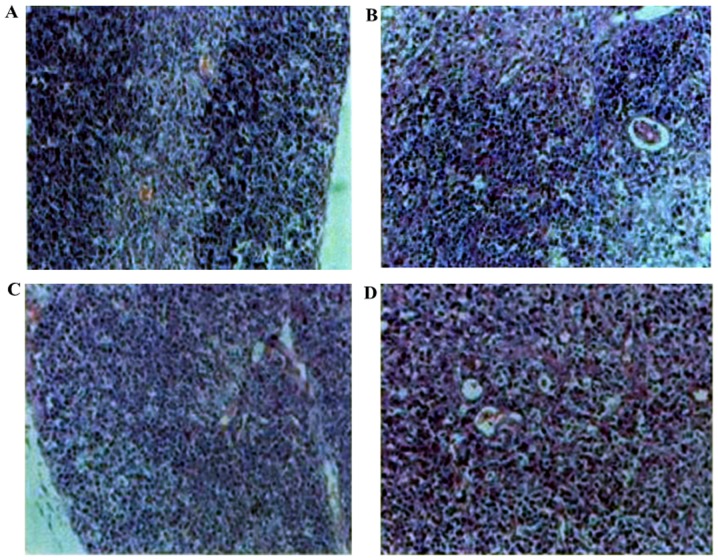
Effect of *Rhodiola rosea* extract on the histopathological changes of the thymus in rats (hematoxylin and eosin staining; magnification, x200). Groups of mice were pre-treated with *Rhodiola rosea* extract and challenged by caecal ligation and puncture for 24 h. Histopathological changes in thymus tissues from rats. (A) Normal control group; (B) sham-operated group; (C) control group; (D) treatment group.

**Figure 19:**
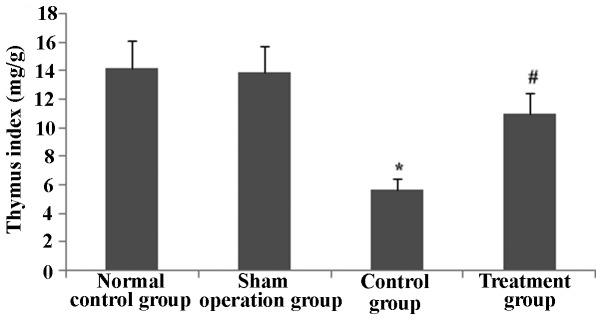
Effect of *Rhodiola rosea* extract on the thymus index of the thymus in rats. Groups of mice were pre-treated with *Rhodiola rosea* extract and challenged by caecal ligation and puncture for 24 h. The thymus index is expressed as the mean ± standard deviation of data from triplicate experiments. ^*^P<0.05 vs. the sham operation group; ^#^P<0.01 vs. the control group.

To evaluate the effects of *Rhodiola rosea* extract on the histopathological changes in the thymus of mice with CLP-induced sepsis, histological assessment was performed of thymus tissue collected 24 h after the administration of CLP with or without treatment. As shown in Fig. 18, the thymus lobular structure disappeared and the cortical and medullary lymphocytes were significantly reduced after the CLP operation. After administration of *Rhodiola rosea* extract, the thymus exhibited lobular structures, and cortical and medullary lymphocytes were significantly increased. The thymus index was also determined. As shown in Fig. 19, the thymus index was markedly lowered at 24 h after CLP (P<0.05), while pre-treatment with *Rhodiola rosea* extract markedly increased the thymus index (P<0.05). Thus, *Rhodiola rosea* had a protective effect on the thymus.

### Rhodiola rosea extract increases the survival of septic mice

**Figure 20:**
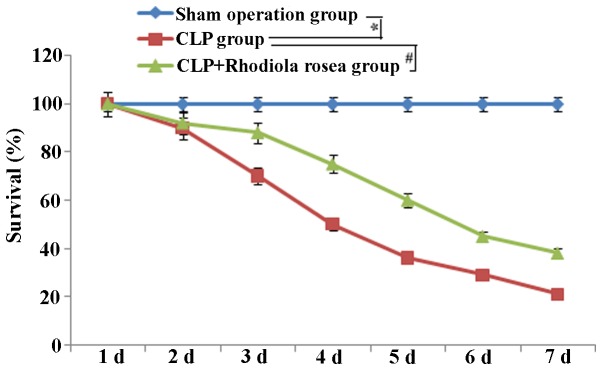
Survival curve. CLP group, group challenged with CLP; CLP + *Rhodiola rosea* extract group, group challenged with CLP and treated with *Rhodiola rosea* extract. ^*^P<0.05 vs. sham operation group; ^#^P<0.05 vs. CLP group. PLP, caecal ligation and puncture. d, day.

The survival of mice was markedly decreased in mice receiving CLP compared to that of mice in the control group. The decreased survival induced by CLP was significantly attenuated by pre-treatment with *Rhodiola rosea* extract as compared to that in the untreated CLP-induced group (Fig. 20).

## Discussion

Several studies have shown that immune disorders are involved throughout the entire occurrence and developmental process of sepsis, which ultimately leads to organ failure and subsequent mortality (26). The regulation of the body’s immunity has long been a focus of biomedical research (27). Sepsis is one of the leading causes of mortality of clinical patients (28). The occurrence and development of sepsis are caused by an imbalance of pro-inflammatory and anti-inflammatory mechanisms (29). When the disease develops to a certain degree, the body’s immune function is inhibited to various degrees, which eventually leads to a highly lethal immune disorder that is characterized by immune cell apoptosis and severe paralysis (30). The large decrease in the number and function of immune cells through apoptosis is one of the major causes of the high mortality in patients with severe sepsis (31,32). Apoptosis of cells in central lymphoid organs (spleen) and peripheral circulating lymphocytes in patients with severe sepsis was shown to be significantly increased, particularly in patients with septic shock, and eventually leads to paralysis which is dominated by a specific immune dysfunction (33). Thus, the reduction of lymphocyte apoptosis is currently one important and challenging aspect of sepsis treatment and an important measure to decrease the mortality rate of patients with sepsis (34). The present study revealed that mice with sepsis demonstrated an increase in thymus T-lymphocyte apoptosis as well as a decrease in the thymus T-lymphocyte count. Furthermore, CD^+^, CD4^+^ and CD4^+^/CD8^+^ T-lymphocyte sub-sets were decreased and the secretion of IL-2 and IFN-γ was decreased, leading to immune suppression in the host.

TNFAIP8 has an important regulatory role in cell apoptosis and signal transduction, as well as tumor occurrence, development and invasion (35). TIPE2, a newly discovered protein of the of TNFAIP8 family, has drawn increasing attention (36). TIPE2 was initially discovered by Sun *et al* (37) at the University of Pennsylvania in an experimental autoimmune encephalomyelitis model and is an essential protein that is important in maintaining immune homeostasis. Sun *et al* (37) found that TIPE2 was associated with caspase-8 and regulated the nuclear factor (NF)-κB pathway via apoptotic enzymes. There is evidence indicating that TIPE2 can inhibit the activation of activator protein-1 and NF-κB, while cells lacking TIPE2 genes demonstrate high reactivity in response to Toll-like receptor and T-cell receptor signaling (38). In addition, in a sepsis-induced mouse model treated with low doses of endotoxin, septic shock occurred in TIPE2-knockout mice (39). However, further analysis has indicated that the downregulation of TIPE2 genes induces persistent lymphocyte activation, enhancement of Fas expression, promotion of lymphocyte apoptosis and increased production of IL-4, IL-6, IL-12 and IFN-γ (37). TIPE2 also functions to regulate cell death, and the expression of inactive TIPE2 genes can inhibit Fas-mediated apoptosis and antigen receptor-induced cell death (40). Moreover, the numbers of T lymphocytes, B lymphocytes and dendritic cells in TIPE2-knockout septic mice were significantly increased (41). Thus, TIPE2, as a newly discovered protein, has an important role in immune homeostasis. The results of the present showed that with an enhancement in TIPE2 expression, the expression of apoptosis-promoting proteins, including Fas, FasL and Bcl-2, also increased T-lymphocyte apoptosis and the T-lymphocyte count, and significantly decreased the CD3^+^, CD4^+^ and CD4^+^/CD8^+^ T-lymphocyte sub-sets as well as immunity. However, these changes were reversed in animals pre-treated with Rhodiola.

In human sepsis, two immunologically distinct stages are observed: An early pro-inflammatory phase and a late, compensatory anti-inflammatory phase (42). CLP-induced sepsis increases lymphocyte apoptosis, which has been shown to cause immunosuppression during the late phase of human sepsis (43). Immunological cytokines are functionally categorised into two groups: One group with Th1-like properties and a second group with Th2-like properties. Activated CD4^+^ T cells are programmed to secrete cytokines with inflammatory properties, including the Th1-type cytokines IFN-γ, IL-2 and IL-12, or cytokines with anti-inflammatory properties, including the Th2-type cytokines IL-4 and IL-10 (44). In addition to attenuating the abrupt release of pro-inflammatory cytokines, including TNF-α, IL-1β and IL-6, treatment with chlorogenic acid enhanced serum Th1 cytokines during the late phase of sepsis without affecting Th2 cytokine release (45). It has been suggested that increases in Th1 cytokines are beneficial in the late stage of sepsis when immunosuppression predominates and can cause mortality (46). Decreased Th1 function has been previously reported in patients with peritonitis, and defective T-cell proliferation and cytokine secretion correlate with mortality. In the present study, CLP facilitated the secretion of Th1 cytokines and Th2 cytokines. Of note, pre-treatment with *Rhodiola rosea* further increased the secretion of Th1 cytokines while not affecting Th2 cytokine levels, and therefore improved the host’s immunity.

The thymus is an important immunoregulatory organ and has an important role in SIRS and MODS. The results of the present study indicated that the apoptotic rate of thymus T lymphocytes increased, the lobular structure of the thymus disappeared, that cortical and medullary lymphocytes were significantly reduced, and that the thymus volume and thymus index were deceased 24 h after CLP. However, in the group pre-treated with *Rhodiola rosea* extract, the apoptotic rate of thymus T lymphocytes decreased, the thymus exhibited lobular structures, cortical and medullary lymphocytes were significantly increased, and the thymus volume and thymus index were increased.

Furthermore, the results of the present study showed that the survival of the mice was higher in the treated group compared with that in the model group. Furthermore, the pathological changes were milder in the treated group compared with those in the model group, and the apoptotic rate was significantly decreased (P<0.05), which demonstrated that *Rhodiola rosea* extract has a protective effect on the thymus by preventing apoptosis. The apoptotic rate was dependent on the TIPE2 status of T cells from the thymus and was decreased in septic mice pre-treated with *Rhodiola rosea*; furthermore, changes in Th1 and Th2 cytokines were also in parallel with changes in TIPE2 expression, which suggested that *Rhodiola rosea* extract can alleviate thymus injury by lowering TIPE2 expression, increasing the secretion of Th1 cytokines, inhibiting thymus T-lymphocyte apoptosis, and increasing the CD3^+^, CD4^+^, CD8^+^ and CD4^+^/CD8^+^ T-lymphocyte sub-sets, ameliorating the host’s immunity and increasing the survival of mice.

In recent years, studies have shown that *Rhodiola rosea* can strongly enhance cellular immunity and humoral immune function in mice, which has become a research hotspot (47). Previous studies have indicated that the extract of *Rhodiola rosea* can inhibit tumor growth in S-180 tumor-bearing mice, potentially via the enhancement of the body’s immune function, thereby inducing the secretion of cytotoxic proteins by T cells, resulting in increases in antibody-secreting B cells and antibodies, thus inhibiting the growth of tumors (48,49). Previous studies have shown that within the dose range of 500–2,000 mg/kg, *Rhodiola rosea* can enhance the relative quality of the immune response, foot pad thickness, thymus weight and spleen antibody production in BALB/C mice (50). Intragastric administration of the stem and leaf extract of *Rhodiola rosea* at 250–500 mg/kg to mice for seven days increased the quality of the immune response in normal mice and significantly enhanced phagocytosis by the reticuloendothelial system (51). Reproduction of these experimental conditions in a rat model with low immune function induced by cyclophosphamide, which was treated with rhodiola polysaccharides from Tibetan *Rhodiola rosea* demonstrated that rhodiola polysaccharides had no significant effect on white blood cells in peripheral blood or the thymus quality, although it lowered the peripheral blood haemoglobin content in normal mice and improved the blood haemoglobin content in immunodeficient mice (52). In addition, *Rhodiola rosea* promoted the differentiation of spleen lymphocytes and the activity of natural killer cells, thus reversing the abovementioned changes in immunosuppressed mice (51). Rhodiola can also strengthen the function of the mononuclear phagocyte system, thereby improving the body’s immune defence (53). In accordance with the results of a previous study (54), the present study indicated that the total glycosides of rhodiola can suppress TIPE2 expression in mice with sepsis, lower the expression of the apoptosis-promoting proteins Fas and FasL, decrease T-lymphocyte apoptosis, increase the number of thymus T lymphocytes and the CD3^+^, CD4^+^ and CD4^+^/CD8^+^ T-lymphocyte sub-sets, increase the levels of Th1 cytokines, including IFNγ, IL-2 and IL-12, and enhance the host’s immunity.

In conclusion, the present study showed that the total glycosides of rhodiola were able to suppress the sepsis-induced overexpression of TIPE2 in mice, inhibit the upregulated expression of the apoptosis-promoting proteins Fas and FasL, increase the decreased expression of the apoptosis-inhibiting protein Bcl-2, decrease the enhanced T-lymphocyte apoptosis, increase the decreased numbers of thymus T lymphocytes and CD3^+^, CD4^+^ and CD4^+^/CD8^+^ T-lymphocyte sub-sets, further enhance the Th1 cytokines IFNγ, IL-2 and IL-12, and enhance the host’s immunity. These findings provided a theoretical basis for the application of rhodiola in the treatment of sepsis.

## Figures and Tables

**Table I:** Primer sequences used for using reverse transcription polymerase chain reaction to validate the microarray analysis.

Gene	Primer sequence	Product (bp)
TIPE2	F: 5′-GGGAACATCCAAGGCAAG-3′ R: 5′-AGCTCATCTAGCACCTCACT-3′	195
Fas	F: 5′-GACCCAGAATACCAAGTGCAAGTG-3′ R: 5′-CTTGCCCTCCTTGATGTTATTTTC-3′	400
FasL	F: 5′-CGTGAGTTCACCAACCAAAGC-3′ R: 5′-CCCAGTTTCGTTGATCACAAG-3′	219
Bcl-2	F: 5′-TTCTCCTTCCAGCCTGAGAGCAA-3′ R: 5′-ATGACCCCACCGAACTCAAAG-3′	317
β-actin	F: 5′-GACTACCTCATGAAGATCCTCACC-3′ R: 5′-TCTCCTTAATGTCACGCACGATT-3′	190

F, forward, R, reverse.

## Abbreviations

TIPE2: TNF-α-inducible protein 8-like 2
CLP: caecal ligation and puncture
Bcl-2: B-cell lymphoma 2
FasL: Fas ligand
Fas: Fas receptor
Th1: T helper type 1 cells
Th2: T helper type 2 cells
IL-2: interleukin-2
IL-10: interleukin-10
TNF-α: tumor necrosis factor-α
IFNγ: interferon gamma
